# The embodied mind in motion: a neuroscientific and philosophical perspective on prevention and therapy of dementia

**DOI:** 10.3389/fpsyg.2023.1174424

**Published:** 2023-08-17

**Authors:** Erik N. Dzwiza-Ohlsen, Gerd Kempermann

**Affiliations:** ^1^Faculty of Arts and Humanities, Husserl Archives Cologne, University of Cologne, Cologne, Germany; ^2^German Center for Neurodegenerative Diseases (DZNE), Dresden, Germany; ^3^CRTD – Center for Regenerative Therapies, TU Dresden, Dresden, Germany

**Keywords:** Alzheimer’s disease – AD, neurodegeneration, plasticity, reserve, exercise, resilience, body-language, environment

## Abstract

The embodied mind in motion is a concept in which health and well-being, prevention and therapy, as well as lifestyle and habits meet. The mind changes profoundly in the course of dementias, affecting daily living and resulting in reduced quality of life. Interdisciplinary approaches are required for a holistic understanding of how the mind is affected by dementia. We here explore what such a holistic theory of dementia might look like and propose the idea of “embodied mind in motion”. The paradigm is biopsychosocial or biocultural, the theoretical anchor point is the lifeworld, and the guiding concept is “embodiment,” as body and mind are constantly in motion. Physical activity is, hence, central for the experience of health and well-being, beyond being “exercise” and “health behavior”. We discuss the embodied mind in motion referring to phenomenology, enactivism and (philosophical) anthropology. In our view, habits are embodied long-term memories and a philosophical equivalent to lifestyle. They unfold the meaningfulness of moving the body, complementing the objectifiable benefits of physical exercise. Empirical studies on “holistic activities” like hiking, yoga, music and dance illustrate improved integration into everyday life. Their meaningfulness enhances compliance and increases the preventive and even therapeutic potential. A crucial factor for this is the emotional dimension of lifestyle, exemplified by the virally popularized performance of “Swan Lake” by wheel-chair bound ex-ballerina Marta Cinta González Saldaña, suffering from Alzheimer’s disease. A number of epistemological and ontological consequences anchor “embodied movement” as a valuable principle for dementia research.

## 1. Introduction: embodied prevention as the silver bullet against dementia?

From an evolutionary perspective, movement is intimately linked to the genesis of nervous systems, which are the necessary condition of higher cognition. Consequently, movement might play a crucial role for the maintenance of nervous systems and cognition. How great this potential actually is can be investigated in cases of neurodegenerative diseases. For this, purely mechanistic conceptions of movement that have dominated medicine to date have to be overcome by establishing a more holistic understanding of human movement.

The simplest operational definition of dementia is the irreversible loss of brain functions. These functions are not limited to higher cognitive functions, such as memory, orientation and language, but ultimately include everything the brain does. Nevertheless, in most contexts dementias are used as an umbrella term for age-related, chronic, progressive, incurable, and, by and large, irreversible symptom complexes that are interpreted as “fundamental disorders of higher-order consciousness” (Fuchs, 2020a, 670). Dementias are no diseases in themselves and the causal factors, clinical manifestations and courses differ vastly. Alzheimer’s disease (AD) accounts for roughly two thirds of all cases of dementia. Given the far-reaching consequences of AD across scales from molecular to social, narrow operational definitions are obviously insufficient as they neglect the complexity of the human condition.

Even within the biomedically originating perspective a *bio-psycho-social paradigm* can already overcome certain conceptual shortcomings: while dementias are the consequences of neurodegeneration at a biological level, there is no strict causal correspondence between neuropathology and the clinical symptoms and manifold consequences of the dementia. Some affected persons might, despite massive neurodegeneration, have relatively low functional losses and due to a supportive social environment live reasonably well with their disease. On the other hand, even with comparatively low neuronal degeneration, functional losses might be great and, especially in challenging social environments, result in a low quality of life. However, the bio-psycho-social framework requires concretization. For care research this has been accomplished for example in *positioning theory* (Sabat and Harré, 1992, 1994) or *person-centered care* (Kitwood and Bredin, 1992; Kitwood, 1997), but not yet for prevention.

Currently, the life sciences and the humanities tend to mark extreme poles in the discourse on the prevention of dementia: On the one hand, evidence-based “checklists” of health behaviors, obtained from large epidemiological studies and meta-analyses dominate the life sciences. While the recommendations by themselves are entirely reasonable and well-grounded, the one-size-fits-all approach results in low compliance and ignores many interaction effects. For example, energy uptake through diet and energy consumption stands in a complex interdependence that can affect risk and course of dementia in many ways. On the other hand, approaches like *New Dementia* (Leibing and Schicktanz, 2021) reflect political, economic, social, cultural, and ethical aspects in epistemic terms. Thus, *New Dementia* offers a critical re-evaluation of the “preventive turn” that questions the evidence, objectivity and neutrality of life science recommendations (Schweda and Pfaller, 2021), but runs at the same time the danger of losing touch with the clinical realities. We thus believe that a clinical definition of dementia is needed as an anchor point for a comprehensive interdisciplinary dialogue. Clinical definitions are necessary for all practical purposes and they must also provide the starting point for our considerations here, because only for definable clinical entities epidemiological and clinical data provide solid grounds to define and appreciate the consequences of prevention.

As a fertile middle ground in a demanding sociohistorical constellation, we propose the interdisciplinary idea of “the embodied mind in motion” (Kempermann, 2022): first, there is to date no causal therapy for dementias; second, demographic change results in disproportionately more old and oldest people, increasing the prevalence of dementia and demand for institutionalized care, for which fewer care-givers will be available. Third, institutional care faces the challenge of exploding costs to stressed social systems and an increasing shortage of specialists. Finally, multimorbidity and polypharmacotherapy increase in old age so that attention to numerous illnesses with a wide variety of treatments have to be coordinated. As a result, the quality of life of people with dementia is reduced not only due to the dementia itself, but also through the many indirect effects on family, caregivers and society.

This situation is not solvable even if the current promising small successes with antibody-based treatments such as Aducanumab and Lecanemab become more widely applicable therapies. Rather, emphasis on prevention is key, especially under a global perspective with massive increases in the incidence and prevalence of dementias in Asian and African countries. However, the full potential of prevention is not yet fully exploitable. For example, the temporal dissociation of necessary early preventive interventions and the evaluation of their success is problematic. The discovery of early biomarkers for dementia are changing this, but are burdened with ethical conflicts, if healthy populations are screened in the absence of true treatment options. Nonetheless, prevention has proven success in terms of dementia-free years and the reduction of symptoms. However, to define the success of prevention merely as the reduced incidence (and prevalence) of dementia symptoms is not sufficient, because in their individual setting, people might be impaired differently by states with comparable biomarker profiles. A more individualized approach is necessary.

In addition, check-lists of recommended health behaviors remain life-less and, despite the proven effects of individual actions, receive too little compliance. As insufficient a simplistic definition of dementia is, as insufficient is a purely mechanistic understanding of prevention that is mainly reactive to key physiological parameters. But lifestyle-based prevention is promising, if it reflects what the individual can do to increase resilience under his or her personal set of conditions.

All phenotypic variation, including the response to preventive strategies, is the consequence of genetic variance and the influence of an environmental factor. Environment, however, encompasses both *extrinsic* influences, e.g., socio-economic, climatic, etc., and *intrinsic* influences, the non-shared environment. The non-shared environment reflects the impact of our individual response to genetic and environmental conditions, including lifestyles, which are highly personal and individual behavioral patterns shaped by experience. While dementias tend to have substantial heritability, both types of environmental factors play an important role. The decrease in the cohort-specific prevalence of AD in the past decades (a relative reduction of roughly 16% per decade) speaks to the fact that the influence of behavior and hence lifestyle matters (Wolters et al., 2020).

Lifestyle is of eminent importance for at least two additional reasons: Firstly, we can actively influence lifestyle ourselves and thus take action in an area that is otherwise characterized by the experience of helplessness; secondly, lifestyle factors are not only directly related to prevention but also to therapy and care, thus truly accompanying an individual over the course of life, through health and disease.

## 2. The embodied mind in motion

“Leading a good life” might be the best way to effective prevention, but what this exactly means has been the subject of thousands of years of human discourse in philosophy and religion. In order to live a good life under the Damocles sword of dementia, reductionistic, physical and actionist perspectives on health and aging must be complemented with a holistic, qualitative and lifeworldly perspective. What is known about the success of preventive measures, especially those based on personal lifestyle, paints an increasingly concrete picture of how “leading a good life” is also healthy and can, under supportive genetic and environmental conditions, lead to resilience in old age and “successful” aging in terms of well-being. Empirical studies on examples like hiking, yoga, music and dance illustrate that activity needs to be “embodied” in everyday life and in a way that is meaningful to the individual in order to unfold its medical potential (Kempermann, 2022).

Especially for chronic, progressive and terminal diseases, the complementary perspective captures on one side the subjective and objective deficits that lead to the diagnosis and on the other the individual and social resources that are required for successful prevention, therapy and care. Moreover, it captures not only physical and mental health but also well-being and, ultimately, *eudaimonia* (Aristotle, 2017). This holistic concept, best translated as “flourishing life”, complements by its long-term orientation the short- and medium-term oriented notion of well-being, while it also counterweights the shortcomings of concepts like successful aging. According to a prevailing reception of successful aging, dementia “appears as a worst-case scenario of later life, the ultimate demise of the rationally planning, autonomous, and accountable self—a process that needs to be prevented by any means” (Schweda and Pfaller, 2021, 207). According to the idea of flourishing life, this is by no means the case. And it by no account follows from a low well-being in dementia that life is no longer perceived as flourishing. For Woopen et al. (2021, 139, our transl.), “a person perceives his or her life as flourishing, when he or she can develop those of his or her characteristics and live values and beliefs that are particularly important to him or her.” The challenge, then, is to balance health, well-being and flourishing life without delegating responsibility for it solely to the individual.

From this, a clear task statement for the prevention of dementia can be derived: Evidence-based recommendations for lifestyle interventions in dementia (Livingston et al., 2020) should thus be complemented with this approach that takes greater account of the values of the patient. Subjectively perceived and biographically anchored meaningfulness is a key to realize sustainable prevention strategies in vital contact with the environment.

Evidence-based medicine recommends “moderate physical activity” or taking 10,000 steps per day as a lifestyle intervention for healthy aging in general (Paluch et al., 2022) and “successful” cognitive aging in particular (Iso-Markku et al., 2022). Worldwide, physical inactivity increases the relative risk for AD with a factor of 1.82 (95% CI 1.19–2.78), low educational attainment as proxy for general cognitive activity by 1.59 (1.35–1.86) (Norton et al., 2014). But both factors, as well as other key lifestyle-dependent measures such as diabetes mellitus, midlife arterial hypertension, obesity, depression and smoking are not independent from each other: they have a large communality of up to 65%. Taking care of one risk factor has an impact on other factors. In addition, there lies an immense preventive power in the interaction effect itself.

Classical reductionistic single-intervention approaches decompose holistic lifestyles into abstract (well-measurable) components, which are used as building blocks for standardized but abstract preventive interventions. But for prevention as a lifelong process, a rigid focus on health benefits in the far future is ineffective. The effects of the intervention on well-being at presence must be taken into account. But orientation towards well-being can be reductionist as well: A high level of well-being in the presence by no means entails a high level of it in the future if, for example, the former consumes the resources required for the latter. Here, only the idea of “flourishing life” offers the necessary orientation. Physical activity, health, well-being and flourishing life now need to be brought into a sustainable equilibrium compatible with what is known to promote what is typically referred as successful aging, given the factors of genetics and environment. The scientific challenge is how this healthy and preventive balance in “leading a good life” today can be captured (and measured) and inform healthy lifestyle decisions without checklists.

Physical activity is the lifestyle factor with the greatest and best documented effect sizes in prevention and it directly interacts with other factors, such as sedentary time, diet, body mass index, tobacco and alcohol use, sleep quality, depression, socio-economic status and others. If levels of physical activity are improved in a holistic manner, combining present and future benefits, chances are good that overall lifestyle is improved at the same time. The interdependencies not only exist at the systemic physiological level (e.g., in energy uptake and expenditure) but also in brain and mind. Exercise, for example, affects sleep, can help to suppress craving for nicotine, and induce changes in dietary behaviors.

The dynamic interplay of part (movement) and whole (lifestyle) explains why holistic lifestyle interventions are likely to have sustainable effects, but also why the barriers to their successful implementation are so high. If the movement practices come along with subjectively perceived meaningfulness, they result in sustainable lifestyles that are associated not only with good health but also with a high level of well-being both in the present and in the future. Subjectively perceived meaningfulness and the related emotions are main drivers for particular actions and comprehensive lifestyles that increase the likelihood of successful implementation and an improved quality of life and well-being.

### 2.1. Premises, hypotheses and traditions

The existing comprehensive studies on holistic activities, such as hiking, yoga, music and dance, suggest that the mutual relationships between body and brain, mind and spirit, and environment and life are key determinants of their effectiveness, certainly subjectively, but most likely also objectively. At the interface of philosophy, cognitive and life sciences, we find important theoretical approaches to rethink the relation of those elements. Against the background of the question of personal identity in dementia, Thomas Fuchs writes: “According to the paradigm of embodied cognition, consciousness is not a pure product of the brain, but is rather a comprehensive activity of the entire organism in relation to its environment […]. In this respect, personhood is a manifestation of the life process of a human organism and it is thereby embodied in the capabilities and activities of the whole body” (Fuchs, 2020a, 667). The idea of the *Embodied Mind in Motion*, as roughly outlined previously (Kempermann, 2022), goes in the same direction within the context of the prevention of dementia. As we want to demonstrate, the *embodied mind in motion model* offers a framework for a comprehensive interpretation of the link between movement, cognition and environment, building upon well-established neuroscientific bases, but with the help of philosophy. To unfold this guiding assumption, we can draw from three complementary philosophical traditions: *Phenomenology*, *Enactivism*, and *philosophical Anthropology*. These share the premise of a correlation or interrelation between subject(s), object(s), and world, that is essentially moderated by the body.

*Phenomenology*, as one of the most influential philosophical currents of the 20th and 21st centuries, offers systematic reflections on topics like consciousness, subjectivity, embodiment, expressivity, personhood, etc. The founder of phenomenology, Edmund Husserl, coined the philosophical *lifeworld* theorem. At the heart of the lifeworld-theorem is the attempt to comprehensively grasp the general structures of subject-relative experience in order to establish its function as foundation and orientation for the sciences (Husserl, 1976, 1993, 2008; Dzwiza-Ohlsen, 2019). The phenomenological tradition has consequently been in a productive exchange with psychopathology, psychiatry and psychology for more than 100 years (Stanghellini et al., 2019). The phenomenological research on dementia will be discussed in more detail below. The neurosciences as an overarching discipline still have some untapped potential to use phenomenological reasoning for its theoretical foundations, although there are already path breaking contributions like “neurophenomenology” (Varela, 1996).

The title of our contribution here explicitly alludes to *The Embodied Mind* by Varela et al. (2016, first published in 1991) as the “birth certificate” of *enactivism*. Due to its roots (Maturana and Varela, 1987) and its scope, enactivism appears even more compatible with cognitive and neuroscience than phenomenology, since here embodied cognition serves as the key to the fundamental and dynamical correlation between *organism* and *environment* (Varela et al., 2016). But because this tradition explicitly tries to bridge the gap between the cognitive sciences on the one side, according to which self and mind are the result of the dynamic interplay between brain, body and environment, and our lived everyday experience on the other side, it stands in a highly productive relationship to phenomenological philosophy (Thompson, 2007; Gallagher and Zahavi, 2012; Fuchs, 2017). After overcoming its initial preconceptions against phenomenology, Thompson (2016, xxii–xxix) explicitly acknowledged that the Husserlian lifeworld-theorem provides the foundation for enactivism in terms of theory of science. Enactivism will be discussed in Section 4 of this paper.

At the beginning of the 20th century, the tradition of *philosophical anthropology* was reshaped by philosophers like Max Scheler, Helmuth Plessner or Nicolai Hartmann in an interdisciplinary manner (Dzwiza-Ohlsen and Speer, 2021; Fischer, 2022). Before Plessner’s major bio-philosophical work appeared in 1928, *The Levels of Organic Life and the Human* (2019), together with biologist Frederik J. J. Buydendijk, Plessner (2019) proposed a theory of the understanding of embodied movements in 1925. This theory can serve as a connection between enactivism and phenomenology. On the one hand, it takes its starting point from the lifeworld perspective, indicated by the modified use of the formula for the lifeworld by speaking of “*natural pre- and extra-scientific understanding of expression*” (Plessner and Buytendijk, 2017, 125; emphasis and all quotes of this text were translated by us), while deepening the reflections on embodiment in intersubjective terms. On the other hand, philosophical anthropology remains in constant dialogue with the natural sciences, so that the biologically accentuated considerations on the organism within the environment can be connected. The anthropological approach will be applied in Section 5.

## 3. The phenomenological response to the loss of identity in dementia: embodied habits as embodied long-term memory

Within the philosophical discussion of dementia, the discourse on the identity of self and person stands out (Hughes et al., 2006; Hydén et al., 2014). The question is often not only whether and how dementia-related illnesses change the identity of a person’s self, but, more fundamentally, about whether there is a progressive loss of the identity towards becoming “quasi-persons” or “post-persons” (McMahan, 2003, 46ff., 55). The phenomenological tradition provides a strong counterweight against a dominant tendency in philosophy to doubt the fundamental identity of self and person for people with (late-stage) dementia due to their loss of autobiographical memory abilities.

In the last two decades, there has been a productive application of the embodiment approach to dementia in phenomenology. These go back to thinkers of both classical phenomenology like Edmund Husserl (1859–1938), Martin Heidegger (1889–1976), or Maurice Merleau-Ponty (1908–1961), those in the social phenomenological tradition, such as Alfred Schutz (1899–1859), as well as from the neo-phenomenological tradition, such as Hermann Schmitz (1928–2021). Current examples can be found under keywords such as *embodied selfhood*/*embodied expressivity* (Kontos, 2005), *intercorporeal expression* (Käll, 2017), *intercorporeal personhood* (Zeiler, 2014), *body memory/embodied personhood* (Fuchs, 2020a), *gestural-communicative action* (Döttlinger, 2018, our translation), *therapeutic atmospheres* (Sonntag, 2020, our translation), *disrupted intercorporeality* (Winniewski, 2022), or *situated expressivity* (Dzwiza-Ohlsen, 2021, 2022).

These phenomenological approaches share three premises:

1. Dementias can be understood not only as neurodegenerative *diseases* of the brain but also as psycho- and socio-degenerative *illnesses* of the whole person within his or her lifeworld. Thereby the phenomenological approach represents a counterweight to the often dominant, naturalistic paradigm, which essentially aims at explaining the phenomena through underlying *causalities*. Instead of investigating dementia with a “naturalistic attitude” (Husserl, 1989, 173), it investigates the lifeworld perspective that is characterized by the “personalistic attitude” (Husserl, 1989, 174). This attitude encompasses the emotion, volition, and cognition of embodied persons who stand in a relation of *motivation* to their socio-cultural lifeworld. Like enactivism, all these approaches share the assumption that “experience is not an epiphenomenal side issue but is central to any understanding of the mind, and accordingly needs to be investigated in a careful phenomenological manner” (Thompson, 2016, xxvii). Thereby the phenomenological method tries to grasp “the structure of lived experience” (Kyzar and Denfield, 2022) and thereby also to facilitate a holistic interpretation of psychopathologies (Stanghellini, 2010). Authors like Fuchs (2020a), Summa (2014), and Dzwiza-Ohlsen (2021) have identified the progressive loss of a meta-perspective as a structural feature of Alzheimer’s disease, which links central symptoms. More precisely, this loss is characterized by the reduced ability to reflectively distance oneself from the present situation in temporal, spatial, and social terms through symbolically mediated knowledge. Since two important sources of autobiographical memory, namely episodic and semantic memory (Tulving, 1972), are diminishing; and these are of great importance for everyday communication, not only intra-, but also intersubjective identity constitution is considerably challenged. This condition has already been famously described by Auguste Deter, the first patient diagnosed by Alois Alzheimer in 1905, with the words: “I have, so to speak, lost myself.”
2. The phenomenological approaches share the assumption that for full comprehension we need to look beyond the deficits. Here, the embodied nature of our existence is a crucial resource, providing the basis for several arguments in the aforementioned discourse, for example against the revocation of personhood in late-stage dementias (Tewes, 2021). First of all, these accounts share the assumption that the body is not just a “vehicle of the mind” (Fuchs, 2020a, 665), but body and mind form an inseparable unity. Secondly, they utilize a fundamental distinction between the “lived body” and the “living body”, with divergent definitions existing. Husserl (1989, 240), for example, differentiates between “Leib”, translated as “Body” with a capital b, and “Körper”, translated as lowercase b “body,” which fits well with the terminology of Plessner and Buytendijk (2017, 80), explaining “Leib” as “lived body” (*lebendiger Leib*) and “Körper” as “living body” (*belebter Körper*). On one side, our *lived body* appears to us within the lifeworld as a double-sided unit, i.e., as an experiencing subject and as an experienced object. Husserl has illustrated this with the example of self-touch (Husserl, 1973, 163). On the other side, the *lived body* becomes the *living body/biophysical body* via naturalistic theoretization in biology and medicine. The living body is the organismic, biophysical side, including the brain as an organ.
3. All these accounts, at least according to our reading, agree explicitly or implicitly that the lifeworld is ontologically primordial. The lifeworld is and will remain the foundation of all sciences, which is why all “scientific models […] are formalized representations of the world” and are “destillations of our embodied experiences as observers, modelers, and interveners” (Thompson, 2016, xxvii). However, this fundamental premise does not conflict with interdisciplinary dialogue between philosophy and the life sciences, but forms the very foundation of such dialogue. Nevertheless, the process of such productive interdisciplinary collaboration in the context of dementia seems to be just beginning. Rather than fighting dogmatic battles, we should remember what Merleau-Ponty had stated in 1945, and 75 years later was quoted in an editorial of The Lancet Psychiatry (2021): “There is no choice between a description of the illness that would give us its sense and an explanation [of the disease] that would give us its cause, and there are no explanations without understanding.” For successful medicine, lifeworldly experience and scientific evidence must complement each other as optimally as possible: For example, one may learn to address the lived body also as a living body through technical measurements of vital parameters to inform the embodied practices and overall lifestyle of his/her daily living (e.g., for a more effective training, therapy or prevention).

As a consequence, the perceived discrepancy between natural science’s and philosophy’s perspective on dementia is less dichotomic than it is often postulated. The phenomenological approach can aid a movement towards a more personalized medicine and extend the focus to include the impact of dementias on more aspects of the life of the affected person than usually addressed and include partners, relatives, caregivers and others. The phenomenological tradition will usually offer a richer and more complete description than the empirical studies. Nevertheless, to unfold its full potential in dementia research, phenomenology must be solidly rooted in the measurable, objectifiable facts. For example, the potential of embodiment to serve as a resource in the context of neurodegenerative diseases is quite different depending on whether we are talking about Alzheimer’s dementia, Parkinson’s disease, or Amyotrophic Lateral Sclerosis (ALS) and which stage of the disease is in the focus.

### 3.1. Habits as embodied long-term memory

According to the philosophical locus classicus, Aristotle’s *Nichomachean Ethics* (2017), habits are the sedimented history of our practices within our social environment. As virtues and vices, they are of central importance for our lifelong pursuit for a flourishing life (*eudaimonia*). Biologically speaking, habits on the other hand are behavioral routines, performed consciously or unconsciously. As such, they are the consequence of associative learning and an expression of a stimulus–response relationship (Robbins and Costa, 2017). Importantly, those habits become detached from the goals to which the action might have originally been directed to. Whereas the reductionistic biological definition thus leaves out important aspects of habits in everyday life (e.g., their value), with philosophy alone we cannot study elementary mechanisms that mediate between organism and environment. We propose a middle ground by the notion of embodied habits and lifestyles from within the idea of the embodied mind in motion.

Embodied habits are emblematic of the unity of body and spirit and prove to be a critical resource in the face of AD. According to Fuchs (2020a, 666) embodied habits can be seen as a kind of individual and implicit memory, “which remains preserved right up to the last stages of the illness, and in which the biographical history of the patient is manifested.” This means that these kinds of habits are an essential “form of memory which from birth on integrates a person’s past into her present bodily constitution” (*Ibid.*, 669). But these habits do not only have a “deep vertical” biographical meaning (such as hobbies, rituals, sports, etc.), but also a “broad horizontal” relevance for everyday routines (e.g., brushing teeth, making coffee/tea, dressing and undressing, walking the dog, etc.). We assume that those embodied habits which are vertically deep and horizontally broad are stable and thus can help to sustain health and well-being in times of instability.

Embodied habits form a tacit background of all actions, feelings, and thoughts. They represent a web of pre- or subconscious relations to oneself and the environment and are familiar to me as an intuitive “I-can” (Husserl, 1989, 266). But embodied habits can lose their tacit character through accidents, disabilities, illnesses, and reflection, so that the lived body may become the living body: For example, if my ankle hurts while walking, an arthritis is diagnosed and specific therapeutic measures are initiated, the physical realm steps into the foreground, the disturbed relationship becomes conscious and the subjective unity and stability of the experience is disturbed.

With Kontos (2005), Fuchs (2020a) and Heersmink (2022) one can differentiate several aspects of embodied habits: the procedural, situational, intercorporeal, expressive, and narrative aspect. We briefly explain these aspects, using the example of walking, before we connect them to the notion of lifestyle in Section 4 and use them in an in-depth analysis of dance in Section 5.

The *procedural* aspect entails more or less simple motor sequences and sequences of actions. These range from elementary functions like walking, to more elaborate basic functions like brushing teeth, and finally to complex patterns of, for example, breakfast routines. Procedural habits tend to be very stable and are to a large extent subconscious. Walking upright requires the seamless and constant integration of multimodal sensory information and the adaptive recall of pre-established motor programs.

The *situational* aspect refers to circumstances that elicit certain behaviors. Below this are mechanisms that attract much attention in the cognitive neurosciences (Keum and Shin, 2019). Responding adequately and efficiently to given situations and for planning ahead, being able to recur to pre-formed “habits” in the sense of triggerable response routines offers massive advantages: The cyclist who escapes the opening car door does so by relying on a pre-configured complex, yet flexible response. Bachelard (1964, 92f.) has illustrated the situation-specific yet flexible familiarity of our lived body with the environment by the example of the house we grew up in. Even after years, we intuitively know at which step of the stairs we have to take a larger step in order not to stumble.

The *intercorporeal* dimension can also be illustrated by the example of walking: We synchronize our gait when we walk together and can reach under each other’s arms when the gait becomes unsteady. Striking examples of this aspect are couple or group dancing, team sports or the “embodied choreography” in busy pedestrian zones, which seem to glide effortlessly close to each other.

Following on from this, the *expressive* aspect captures a (socioculturally variable) familiarity with individual and collective habits. To stay with the example of walking, each person has an individual or even characteristic gait, which can signal mood and state through nuances of variation.

Finally, the *narrative* aspect emphasizes that embodied habits are highly significant to our identity. In our view, our narrative identity (Schechtman, 1996) is composed not only of episodic and semantic memory, but also of embodied memory. As we will detail below in Section 6, a narrative can be told not only verbally, but also non-verbally based on embodied habits, consciously used in acting and dance.

## 4. Neurobiology and enactivism: lifestyle-based prevention, embodied cognition and brain reserves

With regard to their temporal dimension, prevention and therapy seem to describe two fundamentally different strategies: Prevention aims at the (distant) future, the occurrence of unwanted events is to be blocked or at least altered; therapy, on the other hand, responds to something that has already occurred in the present and which must be dealt with as adequately as possible by rehabilitative means in order to shape the (near) future in the best possible way.

In the case of dementia, however, it is an important question, whether (not only from a lifespan perspective) prevention and therapy indeed describe two fundamentally different strategies. With the rise of regenerative medicine and its concepts, being more attentive to the processes than the mere end results, the distinction becomes less sharp than it is often assumed. The reason is not only that rehabilitative strategies as part of a therapy are often identical to preventive measures, and that preventive interventions might have therapeutic effects on existing pathology, there is also a fundamental relationship and co-development of pathology and the physiological response of the “healthy” rest of the body. In chronic disease, and especially neurodegenerative disease, which can run clinically silent for decades, plasticity and pathology are linked in a way that can only be grasped with holistic perspectives. The disease not only destroys the healthy brain, it also provokes responses from the parts that (initially) remain unaffected.

The concepts behind the key terms of neural or brain reserve, cognitive reserve, brain maintenance, resilience, etc. have been developed to capture the potential for compensation that an organism has in the face of pathological cognitive aging, e.g., in the sense of a positive difference between the actual cognitive abilities and those expected from the age of an individual. These ideas arose from the observation that there was no strict relationship between the signs of disease-causing pathology in the brain and the clinical phenotype observed. While such buffering capacities can be measured in groups of subjects, what actually constitutes the “reserve” is highly individual. In the past years, a large international interdisciplinary consortium spearheaded by Yaakov Stern has developed consensus definitions of reserves (Stern et al., 2019, 2020). While there are still open questions and some remaining disagreements, what has become increasingly clear is that reserves to a large part depend on the activity of the individual, based on the genetic predispositions and within the opportunities that the personal environment offers. Against this background, prevention equals the formation, maintenance and usage of reserves.

Reserves, however, must be “enacted.” In so far, the metaphor of a reserve equaling the gasoline reserve tank of a car falls short: the reserve is not a fixed, static and passive entity, but the direct expression of a lived potential and is only maintained through active enactment. By and large the available evidence can be summarized as stating that leading a “more active” life in general and by means of the embodied mind in motion in particular is associated with greater reserves. It is precisely on the ground of this model that reserves, which were initially defined in purely preventive terms, and resources, which were also defined in purely therapeutic terms, meet and merge in a dynamic manner. Practices of individually meaningful movements have a preventive potential because, on the one hand, they help us to build up cognitive reserves; these reserves allow us to selectively activate habitual resources for longer and at a higher level. On the other hand, they help us to establish habitual resources that carry as implicit foundations the way we think, feel, and act. In effect, a wider range of cognitively stimulating behavior can be sustained at a higher level, because a greater breadth and depth of the autobiographically significant past is available to shape the life at present. Embodied reserves can be maintained longer. Since embodied habits can be reactivated to make autobiographical aspects accessible which are not, or no longer, accessible to declarative long-term memory; and since procedural memory is, and embodied habits are preserved in AD, embodied long-term memory is a crucial resource in AD and other dementias. This means that if embodied habits are biographically significant, they should also have a preventive potential.

Additionally, the situational aspect of embodied habits from the perspective of the lived body and its relation to reserves could be discussed in the future in a broader context with regard to the relation of organism and environment by means of the living body. Experimental studies in animals, complemented by human research inspired by them, have demonstrated that exposure to “enriched environments” results in positive effects across functional domains, and it includes structural changes and improves resilience (Nithianantharajah and Hannan, 2006; Kempermann, 2019). This research points to the central role of plasticity, the bidirectionally causal relationship between structure and function in the brain. While the enriched environment paradigm is reductionistic, the intervention itself remains a “black box” and open to interpretation and experimental specification. The beneficial effects (compared to a rather arbitrarily defined baseline) have been robust to a wide range of concrete experimental settings. This points to the existence of an evolutionarily conserved fundamental principle of how activity in the sense of “acting in the world” shapes the brain and with that its potential for life-long resilience.

With plasticity, we have at hand a fundamental mechanism that captures the embodied interaction of brain, body, mind, and environment. From the perspective of evolutionary anthropology, the logic of this link is: the more we move, the greater the cognitive load and potential for experience, the more we actually learn. We have to make decisions more often–from which we learn, which allows us to better anticipate the future. The greater our predictive powers the better our adaptations to the challenges imposed by our environment, including the consequences of our own actions and the actions of others. This circular dynamic is reflected at several levels of the embodied mind in motion. On the level of the living body, it modulates the interplay between sensory input, motor output, and the processing of both. Sensory inputs, including proprioception and balance, provide a constant and massive flow of input from the moving body to the brain. Additionally, rhythms of neural activity resonate with patterns of physical movement. On the level of the lived body, we experience the positive effect of rhythms (of movement, music, etc.) not only on a subpersonal, but also on a personal level, so that its dynamism and adaptivity permeate all levels of the human lifeform and lifestyle as facets of the (non-shared) environment. It is precisely this dynamic interplay between both levels that is explicitly underlined by the central research findings on reserves and resources in dementia.

However, in order to better bring neuroscientific and phenomenological perspectives into dialogue, we would like to propose enactivism as a “bridging theory”. According to the difference between *lived body* (of a subject in the lifeworld) and *living body* (of an organism in an environment), one could utilize the *axioms of enactivism*, as proposed by Evan Thompson, to put the bridge on solid pillars. The first two axioms help us to generalize the findings of the perspectives of neurobiology and evolutionary anthropology on the living body, while the third and fourth axiom help us to integrate the findings from phenomenology and philosophical anthropology on the lived body:

- “First, […] cognition and world are interdependently originated via the living body” (Thompson, 2016, xxvi).
- “Second, the nervous system is accordingly [i.e., as part of the living body] understood as an adaptively autonomous dynamical system.” (*Ibid.*)
- “Third, cognition as sense-making is the exercise of skillful know-how in situated and embodied action.” (*Ibid.*)
- “Fourth, a cognitive being’s world is not a pre-specified, external realm, represented internally by its brain, but is rather a relational domain enacted […] by that being in and through its mode of coupling with the environment.” (*Ibid.*, xxvii).

After the first two axioms have been unfolded in this chapter, the other two axioms will be unfolded and discussed using ex-ballerina Marta Cinta as an example.

## 5. Dance, music, and emotion in dementia: movements of expression in action

There is good, albeit sketchy empirical evidence of the importance of dance, music, and emotion in dementias for prevention, therapy and care.

Meng et al. (2020) offered a meta-analysis on the effects of *dance* on global measures of cognition, executive functions and memory performance. A literature review by Klimova et al. (2017) supports the idea that dancing therapy would have positive effects on cognitive, physical, emotional and social performance in dementia. Nevertheless, the majority of studies are based on a rather reductionist understanding of dance. One exception is the study by Kontos et al. (2020), which emphasized the social, emotional and creative dimension with explicit reference to the concept of *embodied selfhood*. Across disciplines, including phenomenology, dance and music are considered to increase the well-being of people living with dementia (Tewes, 2021, 367) and are of particular importance for creating supportive “therapeutic atmospheres” (Sonntag, 2020).

With regard to the therapeutic significance (of the reception) of *music*, a literature review of Bernatzky and Kreutz (2015) encompasses a whole range of positive effects regarding anxiety, attention, depression, apathy, aggression, delusions, agitation, sleep and eating behavior (short and long-term) memory, self-esteem, social behavior, and quality of life. At the same time, these studies, similar to those on dancing, are written in a therapeutic logic, so that the subjective and intersubjective meaningfulness hardly comes into view. Fitting to the basic idea of the embodied mind in motion, a meta-analysis of three studies described a 60% reduction in the risk of dementia when playing a musical instrument (Walsh et al., 2021), while a cohort study on the effect of the frequency of playing music in mid-life on cognition later in life found that the most active musicians had 80% greater odds of being in the top cognitive decile. Musicians suffering from Alzheimer’s disease supposedly keep their skills for a long time, sometimes even remaining able to learn some new pieces. Phenomenological reasoning based on empirical findings suggests that “implicit musical memory (for example developing a liking for melodies heard repeatedly) is preserved much longer than explicit memory for melodies” (Baird and Samson, 2009; Fuchs, 2020a, 671).

With regard to the role of *emotions* in dementia, emotional abilities are retained longer than cognitive ones (Tölle and Windgassen, 2012, 303; Summa, 2014, 484; Fuchs, 2020a, 672). It is widely assumed that persons with dementia can still perceive everyday situations in an emotionally differentiated way and express their emotional state non-verbally, almost regardless of the stage (Deutscher Ethikrat, 2012, 26). So, presumably, musical and emotional skills of persons with dementia are also retained longer than verbally mediated ones. As music can directly speak to both the emotional brain and to the intellect and has a strong social component its fundamental spiritual significance is comprehensible. Additionally, there is a remarkable transfer of positive effects for those engaging in musical training and listening to music (Matziorinis et al., 2022).

### 5.1. The case of Marta Cinta

As the example of Marta Cinta González Saldaña illustrates, there are fundamental connections between music, dance and emotion on the one hand and embodied habits and lifestyles on the other. Marta Cinta has become posthumously famous with a video published on *YouTube* by the NGO “Música para Despertar” (2023).^1^ What we know about the ex-ballerina today is that she dedicated her life to ballet, ran a ballet school in Madrid and that her passion for ballet was known to the people around her. She tried to inspire people for ballet, so that she even posed with ballet students from Valencia in the year before her death in 2020 (Süddeutsche Zeitung, 2023).^2^

In the video of 3 min length, Marta Cinta—sitting in a wheelchair and with an advanced stage of AD—performs movements from a choreography of Peter Tchaikovsky’s *Swan Lake* that she had danced decades ago. Within the terminology offered by the notion of embodied habits, the music was able to activate her procedural and situative long-term memory. The awakened performance conveys high emotional significance through the rich expressive character of her lived body in motion (i.e., the combination of posture, gesture, mimic and gaze), up to a climactic point, when music, movement and expression are in perfect synchronicity. As much as this was still possible to her, Cinta “analyzes” her choreography afterwards and expresses the intense emotions to her caregiver. What is striking to the observer is that her whole appearance in this moment appears to fully embody the attitude, habitus and lifestyle of the ballerina she had been. Or, to put it another way, Marta Cinta actualizes her narrative identity through performance.

This “performance” is highly moving for the viewers, as evidenced by the high click rates that the video attracted. *Prima facie*, the key question seems to be, to which extent what we see are the unconscious traces of past encompassing memories of both motoric programs and cognitive and emotional contents. Is the performance a reflection of a persistent understanding that just cannot be communicated by other means any more, or has it become an emptied vehicle? Against this kind of (plausible but epistemologically problematic) parallelism/representationalism, Merleau-Ponty’s (2005, 209) reflections on the performative nature of speech allow a change of perspective:

We must recognize first of all that thought, in the speaking subject, is not a representation, that is, that it does not expressly posit objects or relations. The orator does not think before speaking, nor even while speaking; his speech is his thought. In the same way the listener does not form concepts on the basis of signs. The orator’s “thought” is empty while he is speaking and, when a text is read to us, provided that it is read with expression, we have no thought marginal to the text itself, for the words fully occupy our mind and exactly fulfill our expectations, and we feel the necessity of the speech.

We could make a similar argument with regard to Marta Cinta’s performance. It is not at all the case that cognitive and emotional contents that are represented internally are expressed by embodied movements externally, but rather the embodied performance is the medium of emotional and cognitive content, whose validity we have no reason to question. Performativity, then, is a non-verbal mode through which our narrative identity can potentially unfold. However, this does not exclude the possibility of evaluating the experience and its meaning by means of verbal language. Marta Cinta’s documented words to the caregiver are living proof of this argument: first, that she felt strong emotions while performing; second, that this performance is highly relevant to experiencing and nurturing her personality; and third, that despite Alzheimer’s, she was still able to perform verbalized evaluation.

Even if these conclusions remain speculative after the singular example, we can still on the basis of empirical findings assume that a high and regular activation of individually meaningful and familiar bodily practices have a great chance of benefiting general cognitive abilities and beyond. Furthermore, building reserves of this kind obviously also means building such a reservoir of experience to be drawn from, when declarative memory and language abilities are already failing. In short, embodiment stands for a fuller notion of memory than what can be put into words.

In some sense, this insight is related to the important yet somewhat blunt notion that for care and therapy, the individual, not the population mean, matters. The reference point becomes intrinsic (even though it includes the social relations) and is not objective evidence derived from large numbers of subjects. Such objectifiable evidence is important for diagnosis and the understanding of the overall condition, but not so much for the appreciation of the individual in its situation.

Remarkably, we can connect these considerations of embodied habits with the concept of lifestyle even from within the phenomenological perspective: Husserl already reflected on the “style of life in affection and action, with regard to the way he [/she] has of being motivated by such and such circumstances. […] The style is […] something permanent, at least relatively so in the various stages of life […] As a result, one can to a certain extent expect how a man [/woman] will behave in a given case” (Husserl, 1989, 270). Admittedly, this notion of lifestyle differs from the definition used in contemporary evidence-based approaches in medicine. However, this is not necessarily a disadvantage: After all, lifestyles entail individually meaningful ways of embodied movement practices, which can be better understood with the help of phenomenology, i.e., exploring its subjective and intersubjective experienced meaningfulness. Potentially, a phenomenologically enriched notion of lifestyle could (help to) bridge what we have called a “subjectivity gap” (Kempermann, 2022) in intervention studies, i.e., the gap between subjectively perceived quality on the one hand, e.g., the joy or dislike of exercising, and the objectively measured quantity, e.g., the VO2max., on the other.

The tacit nature of habits is a good example of how the quality of experience may noticeably change without us being able to clearly articulate this change or measure it. Along the general theory of habits by Husserl, affective (life) styles and habits have the following four features: (1) They shape the way what and how we perceive, judge, and value. (2) They can become attitudes, like in optimism or pessimism, guiding the way we evaluate, judge and decide. (3) They can become embodied in movement, expressivity, clothing, etc. And (4), they are crucial for intersubjectivity, especially empathy, allowing the anticipation of personalities and communities.

In Marta Cinta we might see an ideal example of how an individually meaningful and lifelong cultivated embodied practice fulfills these features. We can assume that ballet has profoundly shaped the way Marta Cinta (1) perceives, judges, and evaluates (including but not limited to, what music is particularly meaningful to her and touches her emotionally); (2) what she evaluates, expects, and discovers according to what criteria (e.g., the critical evaluation of a ballet performance by her former students); (3) how she dresses, makes up, does her hair, and acts (in order to further correspond to her ideal of a ballerina at an advanced age); so that (4) those around her know which activities, topics, and manners she values most. In other words, she literally embodies the subjective component of a life with dance, which can never be captured by the objectifiable effects of taking up dancing as a health behavior. From a scientific, medical, or public-health point of view, the implication obviously cannot be that everybody has to become a ballerina in order to obtain these benefits. Rather, the challenge lies in the fact to empower people to develop their own “Cinta-potential” within and through their lifeworldly body–mind activities. Which body–mind activities can concretely elicit this potential will vary greatly between individuals. The potential lies in anything that keeps the profound link accessible, so that it becomes the foundation of preserved quality of life. Items on prevention checklists do not tend to do this.

We emphasized in Section 3 that embodied habits bear a “deep vertical” significance for personal identity and “broad horizontal” significance for everyday ability. The example illustrates that the lifelong devotion to an embodied, highly meaningful practice formed an intra- and intersubjective identity (deep verticality), which is of huge relevance for carrying through everyday life, especially one would assume, in the reductions of care environments (broad horizontality). The case of Marta Cinta makes us understand that personal identity is deeply rooted in the integrity of the relationship between body, mind and environment. Herein it matches exactly with the third axiom of enactivism, deeply changing the way we understand human cognition: “Third, cognition as sense-making is the exercise of skillful know-how in situated and embodied action” (Thompson, 2016, xxvi). Especially in late-stage dementias, this embodied perspective is of importance for the preservation of identity: “To be embodied means to be situated and oriented towards a field of experience as this body, as *this* history, *this* point of view; and *this* unique personal orientation conveyed by the lived body still exists” (Fuchs, 2020a, 670). With this in mind, Marta Cinta’s example is symbolic of what might be said, echoing the famous words of Auguste Deter: “I have, so to speak, preserved myself.”

These considerations make it clear that, on the one hand, personal identity–also in the case of Auguste Deter–can never be completely lost due to the embodied and embedded nature of cognition. On the other hand, neurodegenerative diseases, such as Alzheimer’s dementia, affect higher cognition to such an extent that those affected can no longer follow the path of identity formation by means of their declarative memory. This is exactly what Auguste Deter expressed and Marta Cinta will not be exempt from this either. Admittedly, the situations in which the ballerina’s lifestyle unfolds do not coincide with her life as a whole, although in this case they permeate each other particularly strongly on the basis of her embodied and embedded narrative identity. Therefore, in the sense of broad horizontality, it can be assumed that the ability to cope with everyday life is maintained at a higher level by both before and after the onset of symptoms. Finally, these considerations are vital for an interdisciplinary dialogue: it is not just about personal identity (which is what philosophers typically focus on) nor just about functional preservation (which is what medicine focuses on).

To sum up, while the unity between brain, body, mind, and environment can never completely dissolve, neurodegenerative diseases profoundly alter the integration of these elements. The embodied mind in motion model helps to capture the essence of this relationship, its endangerment by dementia and its potential for preservation through leading “a flourishing life.”

## 6. The epistemological and ontological shift of the *embodied mind in motion*

By recognizing the expressive dimension of the embodied mind in motion within our sociocultural lifeworlds, the ground is taken away from numerous intrusive dualisms. Embodied expression is fundamental for communication and interaction. At this point, however, there is a gap. On one hand, non-verbal expression in dementia receives increasing attention, given the loss of verbal expression in the course of the disease, on the other hand, there is a lack of theories that capture embodied expression as we experience it in our social interactions in the lifeworld. This gap has already been addressed by an interdisciplinary essay from the biologically oriented philosopher and sociologist Helmuth Plessner and the philosophically oriented biologist and physiologist Fredrik J. J. Buytendijk, which appeared in 1925.

The achievement of Plessner and Buytendijk lies in a theory that unfolds the intersubjective dimension of the lived body from a lifeworld perspective. The lived body creates an “intersphere” (Plessner and Buytendijk, 2017, 88) between person and sociocultural environment which is also characterized as a “sphere of behavior” (*ibid.*, 129). This sphere is indifferent to dualisms like “sensuality and spirituality, physis and psyche, objectivity and subjectivity” (*ibid.*, 89). From a lifeworld perspective “we do not perceive a body-object whose movements lead us to infer an ‘inhabitant’ hidden in the brain like in a capsule” (Fuchs, 2020a, 667). Embodied movements are a means of lifewordly communication and interaction sui generis before these dualisms arise.

This does not mean that we would always immediately understand the respective *content* of the embodied movements, but rather that this always requires a context in each situation (Plessner and Buytendijk, 2017, 125–129), varying, for example, with regard to age, culture, or species. More fundamentally, the approach acknowledges that we are in a direct communicative relation with our social environment by the *mode* of embodied movements. Embodied movements are characterized by “sensefulness and understandability” (*ibid.*, 81) articulated as “body-intentionality” (*ibid.*, 122). Finally, body-intentionality realizes itself between the poles of “movement of action” and “movement of expression” (*ibid.*, 91) in a constant interplay with a socio-cultural environment. This is why the authors (*ibid.*, 79) speak about the “environmental-intentionality of the lived body.” In dementia, this intersphere probably fades much later than the ability for verbal expression.

This means that we rather immediately understand the meaning of the lived body’s expression. If a person limps and contorts the face in pain, we do not reflect at length on the mental state on which this expression is based but simply respond by helping. Nobody would question the trueness of the emotion in a laughing or crying infant. This intuition should also guide us in the case of adult persons with communicative limitations.

Looking at Marta Cinta from this perspective we do not have to ask ourselves what she might actually perceive, feel and think. We immediately grasp the sense of the embodied movements, when she begins by undulating her hand in resonance with the music, several times raises her fingertips quickly and slightly towards her caregiver to turn the music louder. Her head drops in discouragement, when she does not seem to “get in”. The caregiver gently clasps her hand and kisses it so that, thus encouraged, she begins her performance. This all only makes sense in the situation of performing and of being seen by others, and probably no one would doubt that these movements are carried by the sense of a choreography.

It is precisely this familiarity with embodied expression on all sides of the involved parties that is Kontos’ (2005) crucial argument to overcome dualistic, reductionist and constructivist concepts in the discussion of dementia. The example of Marta Cinta demonstrates that in “contact with others, bodily modes of expression and behavior become more important than cognitive powers and the mostly diminished or fragmented speech acts” (Fuchs, 2020a, 670).

Not acknowledging this lifeworld perspective also results in epistemological dualisms. Either the *body–mind* problem arises, i.e., how between a thinking substance (res cogitans) and an extended substance (res extensa) can be mediated. Within this logic, embodiment appears as a “connecting cable between subject and object” (Plessner and Buytendijk, 2017, 113), which is why we then need complicated theories to understand the nature of the “cable” (for example, through theories of simulation, representation, or empathy). This classical dualism provides the background of what enactivism tries to overcome, as the fourth *axiom of enactivism* expresses: “a cognitive being’s world is not a pre-specified, external realm, represented internally by its brain, but is rather a relational domain enacted […] by that being in and through its mode of coupling with the environment” (Thompson, 2016, xxvii).

However, as Fuchs (2020b) has defined the “lived body” as experienced from the first-person perspective of the subject, and the “living body” as object from the third person perspective, the dualism might now become the *body–body* problem (Hanna and Thompson, 2003), i.e., how lived body and living body can be mediated. In contrast, we assume with Plessner and Buytendijk that in our lifeworldly communication and interaction we constantly switch back and forth between first-personal and third-personal perspectives on the ground of the second-personal perspective. Contrary to what Fuchs (2020b, 2f.) suggests, the example of the physician’s attitude toward the patient cannot be reduced to a mere objectivation from a third-person perspective; more precisely, a visit to the doctor illustrates that all perspectives—lived body/living body, first-personal/third-personal, personalistic/naturalistic—constantly intertwine and inform each other within the framework of a dialogical second-person perspective. Or in other words: The lived body is to be thought intersubjectively in the face-to-face-encounter. As a result, the lifeworld approach can lead to a fundamental change in perspective which is of high relevance for our communication and interaction with people with dementia in particular and communicative limitations in general. It can also inspire more realistic frameworks for health behaviors, prevention, care and therapy.

## 7. Discussion and perspective: a call for interdisciplinary movement research

In this article we have brought phenomenological and neuroscientific perspectives into dialogue, revolving around the embodied mind in motion. In light of dementia being one of the significant global challenges, we thoroughly examined and discussed the vital role of the embodied mind in motion in promoting both preventive and therapeutic benefits for health, well-being, and a flourishing life.

Although there is now well-established evidence for the enormous effect of moderate physical activity on health in general and successful aging in particular, the potential of this prevention strategy can only be unfolded if it is incorporated into the lives of the people as literally a true lifestyle in the sense of how to live a good life. This need for subjective meaningfulness as a main factor with regard to the probability of enduring implementation of health behaviors is indicated by empirical studies on holistic activities like hiking, yoga, playing a musical instrument, and dancing. Such evidence is deepened by phenomenological reflections on embodied habits and linked to the philosophical question of identity in progressive dementia. Moreover, plasticity as the structural relation between body, mind and environment offers mechanistic insights into the dynamics of embodied prevention. The *embodied mind in motion model* offers a framework for a comprehensive interpretation of the link between movement, cognition and environment, building upon well-established and -studied neurobiological bases, but with the help of phenomenology, enactivism, and anthropology beginning to conceptually fill the black boxes of, for example, the studies on environmental enrichment. In addition, we suggest that a phenomenologically enriched notion of lifestyle as embodied habits could be able to bridge any “subjectivity gap” in lifestyle intervention studies. Finally, the emotional dimension of embodied habits in general and embodied expressivity in particular and its significance for therapy and care was unfolded using the example of the ballerina Marta Cinta. With regard to expressive movements, non-verbal expression has to be researched much more intensively than has been the case so far (Döttlinger, 2018). New measurement techniques and settings have to be used or developed, e.g., using eye tracking, video recordings, wearables or smartphone-based sensors. Furthermore, a reevaluation of institutional care is imperative, aligning with the “transformational shift” proposed by Kontos (2005, 565). The prevalent health-oriented model of institutional care, which often creates a discord between freedom and security, needs to be expanded to incorporate a eudaimonistic-oriented approach. Such a model would provide the essential framework for individual needs, desires, and values to flourish through personally meaningful movement practices within their sociocultural environment.

However, it is important to point out some limitations of the philosophical concept of embodied habits as a resource: first, since these embodied resources also rely on the structural integrity of the brain, the potential of this resource, too, gradually diminishes in later stages of dementia-related illnesses; second, it also becomes clear (again) that conceptual considerations and empirical findings must complement each other. Although the findings presented here have a high face validity for Alzheimer’s dementia, this approach would still need to be evaluated with regard to other neurodegenerative diseases, such as Parkinson’s, frontotemporal dementia or ALS as well as to secondary neurodegeneration after stroke or infection. The degree to which physical integrity and cognitive performance may be decoupled is particularly impressive in ALS, where in the locked-in state communication and expression is reduced to eye movements. As long as the ALS patients do not suffer from cognitive deficits, which happens in certain forms of the disease, they still maintain a rich inner life and even a surprisingly large quality of life. This subjective quality of life tends to be greater than estimated by the care-givers and relatives (Aust et al., 2022). Thirdly, the preventive perspective continues to be of fundamental importance, but the central task at the level of individual biographies is to develop the Cinta-potential and thus to build up reserves in a targeted manner. This article offers some pointers to how this could be done more successfully.

## Author contributions

All authors listed have made a substantial, direct, and intellectual contribution to the work and approved it for publication.

## Conflict of interest

The authors declare that the research was conducted in the absence of any commercial or financial relationships that could be construed as a potential conflict of interest.

## Publisher’s note

All claims expressed in this article are solely those of the authors and do not necessarily represent those of their affiliated organizations, or those of the publisher, the editors and the reviewers. Any product that may be evaluated in this article, or claim that may be made by its manufacturer, is not guaranteed or endorsed by the publisher.

## Footnotes

^1^ https://www.youtube.com/watch?v=owb1uWDg3QM&t=22s

^2^ https://www.sueddeutsche.de/kultur/marta-cinta-ballerina-demenz-1.5115417
